# Contribution of males to brood care can compensate for their food consumption from a shared resource

**DOI:** 10.1002/ece3.6150

**Published:** 2020-03-04

**Authors:** Eva M. Keppner, Manfred Ayasse, Sandra Steiger

**Affiliations:** ^1^ Institute for Evolutionary Ecology and Conservation Genomics Ulm University Ulm Germany; ^2^ Department of Evolutionary Animal Ecology University of Bayreuth Bayreuth Germany

**Keywords:** biparental care, burying beetle, *Nicrophorus*, parental care, sexual conflict

## Abstract

The sharing of the same food source among parents and offspring can be a driver of the evolution of family life and parental care. However, if all family members desire the same meal, competitive situations can arise, especially if resource depletion is likely. When food is shared for reproduction and the raising of offspring, parents have to decide whether they should invest in self‐maintenance or in their offspring and it is not entirely clear how these two strategies are balanced. In the burying beetle *Nicrophorus vespilloides*, parents care for their offspring either bi‐ or uniparentally at a vertebrate carcass as the sole food source. The question of whether biparental care in this species offers the offspring a better environment for development compared with uniparental care has been the subject of some debate. We tested the hypothesis that male contribution to biparental brood care has a beneficial effect on offspring fitness but that this effect can be masked because the male also feeds from the shared resource. We show that a mouse carcass prepared by two *Nicrophorus* beetles is lighter compared with a carcass prepared by a single female beetle at the start of larval hatching and provisioning. This difference in carcass mass can influence offspring fitness when food availability is limited, supporting our hypothesis. Our results provide new insights into the possible evolutionary pathway of biparental care in this species of burying beetles.

## Graphical abstract

The evolution of biparental care is not always well understood. Here, we show that a male's contribution to brood care can compensate for its own food consumption from a resource shared among all family members.

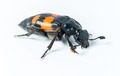

## 1 INTRODUCTION

Life together in family‐like associations leads to a variety of conflicts, with the sexual conflict over the amount of parental care provided and the conflicts between parents and their offspring being two of the better known (Kilner & Hinde, 2012; Lessells, 2012; Trivers, 1974). One facet that both of these conflicts have in common is the conflict over the allocation of resources between the respective members of the family (Kramer et al., 2017; Pilakouta, Richardson, & Smiseth, 2016; Trivers, 1972). Especially in species in which parents provision their young, a struggle to obtain more food than other family members is not uncommon and parents have to balance self‐investment and investment in their progeny (Kramer & Meunier, 2018; Smiseth & Royle, 2018; Trivers, 1972, 1974). A main focus in this field has been directed toward biparentally caring birds that need to collect suitable food within a foraging territory and to decide between feeding it to their offspring and eating it themselves. For example, a study on barn swallows has shown that caring parents do not sacrifice their own nutritional demands during harsh foraging conditions but only increase their caring workload under good conditions, thereby balancing self‐investment and investment in their offspring (Schifferli et al., 2014). If offspring are capable of foraging themselves, competition for food between parents and offspring might arise and can even hamper the evolution of family life (Kramer et al., 2017).

The sharing of a limited food source such as dung or carrion between all members of a family, as is observed in various genera of invertebrates, might intensify this scenario (Smiseth & Royle, 2018). However, the benefits that are connected to reproducing at a shared resource also foster the evolution of family life around these feeding sites. Since parents do not have to forage in distance and thereby leave their brood vulnerable to predation, they can care for their brood while simultaneously nourishing themselves and hence increase the chances of investing in future reproduction (Chemnitz, Bagrii, Ayasse, & Steiger, 2017; Creighton, Heflin, & Belk, 2009; Kramer & Meunier, 2018). Use of a valuable food source for reproduction might additionally entice a male parent to stay with a brood instead of leaving it and might be a driver for the evolution of biparental care. However, can parental care direct toward offspring compensate for the reduced amount of nutritional resources? To date, we have little information regarding the balance between the costs of less food being available for a brood because of more consumers occurring at the food source and the benefits of more care being given by a second parent.

**Figure 1:**
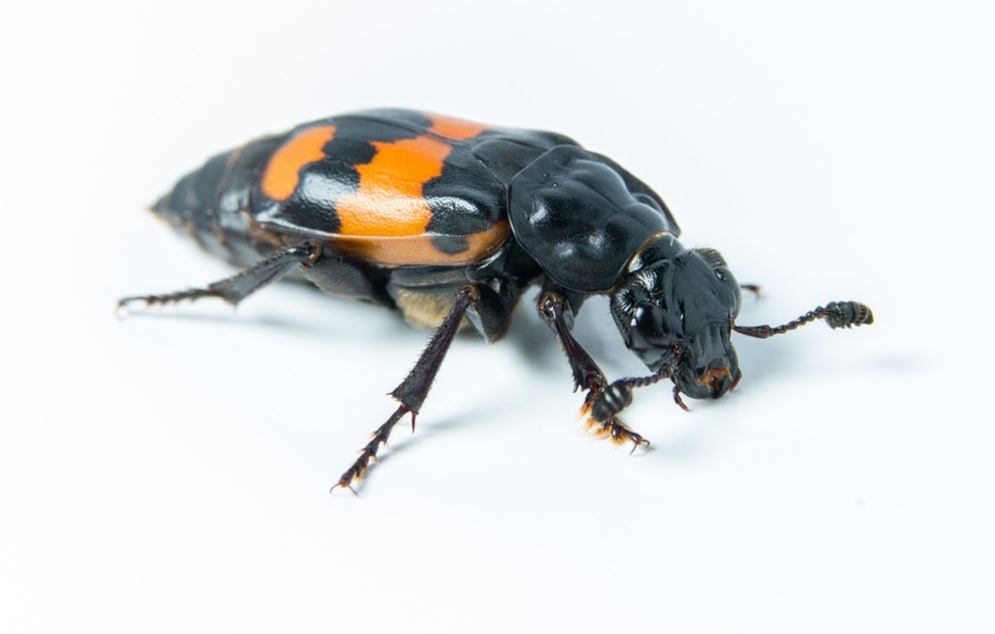
A male of the burying beetle *Nicrophorus vespilloides*

Burying beetles of the species *Nicrophorus vespilloides* (Figure 1) represent a well‐suited organism for investigating the relationship of food consumption from a shared resource, the caring for a brood and the way that this relationship might contribute to the evolution of biparental care. These beetles perform elaborate pre‐ and posthatching parental care to improve the survival and fitness of their offspring, with small vertebrate carcasses being the exclusive food source for both parents and offspring during the duration of reproduction (Eggert & Müller, 1997; Pukowski, 1933). Even though most broods are reared by a male and female pair, both sexes are able to care for the brood alone and can cover the whole range of necessary parental behaviors. Surprisingly, multiple studies have shown that broods thrive equally well if reared by either both or a single parent on same‐sized carcasses (Bartlett, 1988; Müller, Eggert, & Sakaluk, 1998; Smiseth, Dawson, Varley, & Moore, 2005), and if one parent deserts the brood or dies, the remaining parent is able to care for the offspring successfully. If biparental care does not have a conspicuous positive effect on offspring fitness compared with uniparental care, why has biparental care evolved in this species in the first place? Male burying beetles usually do not stay with the brood as long as the female and leave their brood already before larvae disperse (Eggert & Müller, 1997; Scott, 1990; Scott, 1990; Ward, Cotter & Kilner, 2009). Indeed, it already has been shown that females can benefit from this early desertion of male burying beetles in terms of life span (Boncoraglio & Kilner, 2012), probably because there is less competition for the carcass meal (Keppner, Ayasse, & Steiger, 2018). However, a recent study has shown that male burying beetles benefit from staying with the family, because it gives them the opportunity to feed from the carrion resource, thus making them more attractive to the opposite sex after the period of family living (Chemnitz et al., 2017). However, although this personal fitness advantage might be the reason that males prolong their stay, it does not explain why males, despite investing slightly less in care than the females (Capodeanu‐Nägler, Eggert, Vogel, Sakaluk, & Steiger, 2018; Parker et al., 2015; Smiseth et al., 2005), engage in all forms of care such as carcass preparation and offspring provisioning while caring for their offspring together with a female partner (Pukowski, 1933; Scott, 1998). For example, Capodeanu‐Nägler et al. (2018) observed pairs for only one hour, and even during this short period of time, the majority of male *N. vespilloides* were seen to feed their offspring, a result which clearly shows that males participate actively also during posthatching care. Indeed, a recent study with a novel experimental approach has revealed that caring biparentally entails a synergistic effect regarding offspring fitness compared with uniparental conditions (Pilakouta, Hanlon, & Smiseth, 2018). Within this study, the authors maintained the amount of resources and the brood size equal per caring beetle and were thereby able to show that a single parent caring for 15 larvae was less effective compared with two parents caring for 30 larvae on a double‐sized carcass.

The reason for the seemingly contradictory findings in the abovementioned studies of whether there is a positive effect of biparental brood care or not, might lie in the amount of carcass the male burying beetle consumes from the resource while staying with his brood. We hypothesize here that males do indeed contribute to a higher fitness of offspring in various ways but that this effect is masked in experiments that compare uni‐ versus biparental care at same‐sized carcasses, as males not only help to raise the brood but also represent another mouth to feed at the limited food source. In fact, in an earlier study, we found that both parents self‐feed from the carcass, even more when they are in bad condition (Keppner et al., 2018). Thus, the sharing of a limited resource during reproduction can sometimes lead to food competition among family members in the burying beetle *N. vespilloides*.

Hence, the main aim of our study was to reveal whether male care in *N. vespilloides* has a benefit for offspring fitness when the male assists the female parent during care, but this effect can be counteracted by the male's own food consumption. We predicted that carcasses prepared by pairs of beetles, rather than by one beetle alone, would lose more weight during the phase of carcass preparation because of the higher rate of feeding. We tested this by comparing the weight of carcasses prepared by a beetle pair or by a single female. In a second step, in order to disclose the males’ possible positive contribution to care, we attempted to reverse the effect of food consumption by switching the carrion resource of male–female pairs and single females shortly before the larvae hatched. If male care had a positive effect, we predicted that this scenario would lead to a noticeable effect on larval fitness, with a larger brood mass and/or more surviving larvae in the biparental than in the uniparental group. Furthermore, to create scenarios of different resource availability and therefore different intensities of food competition among the family members, we used two carcass sizes, namely large (22 g) and small (5 g), and kept the initial brood size on each carcass size constant.

Those studies that found equal brood success for uni‐ versus biparental care used mouse carcasses of approximately 10 g (Bartlett, 1988), 21 g (Smiseth et al., 2005), and 25 g (Müller et al., 1998). Hence, to be able to draw conclusions also from our small 5g carcasses, we performed an additional experiment to confirm that equivalent brood success in uni‐ and biparental broods also applied in this case.

Finally, we additionally used our experimental setups to measure beetle weight change as an additional fitness parameter for a comparison of the fitness costs of uniparental females, biparental females, and biparental males. We expected uniparental females to gain less weight over the course of the breeding event than biparental males or females, as they are unable to share their parental workload and might have to invest more energy in parental care.

## 2 MATERIALS AND METHODS

### 2.1 Origin and maintenance of the beetles

Experimental *N. vespilloides* were descendants of beetles collected from carrion‐baited pitfall traps in a forest near Ulm, Germany (48°25′03″N, 9°57′45″E). All beetles were maintained in temperature‐controlled chambers at 20°C under a 16:8 hr light:dark cycle in plastic containers (10.0 x 10.0 x 6.5 cm) filled with moist peat and were fed with decapitated mealworms (*Tenebrio molitor*) twice a week. All beetles used in this study were 18–21 days old and had never reproduced prior to the experimental procedures.

### 2.2 Experimental procedures

#### 2.2.1 Switch experiment

To test our hypotheses, we randomly paired unrelated males and females by placing them together in a plastic container (10.0 × 10.0 × 6.5 cm) half‐filled with moist peat. After 24 hr during which the beetles had the chance to copulate, we divided the pairs into two groups: one biparental group in which the male and female were left to care for their offspring together and one uniparental female group where we removed the male. We provided both groups with freshly thawed mouse carcasses. We conducted our experiment with 5 g (5.23 ± 0.25 g) carcasses and with 22 g (22.14 ± 0.45 g) carcasses. Both sizes fall within the typical range used by *N. vespilloides* (Hopwood, Moore, Tregenza, & Royle, 2016; Müller, Eggert, & Furlkröger, 1990; Steiger, 2013). Carcasses within the two size ranges did not differ between the uni‐ and biparental treatments (GLM: 5 g mice: *F*_1,38_ = 0.51, *p* = .48; 22 g mice: *F*_1,36_ = 0.24, *p* = .63).

**Figure 2:**
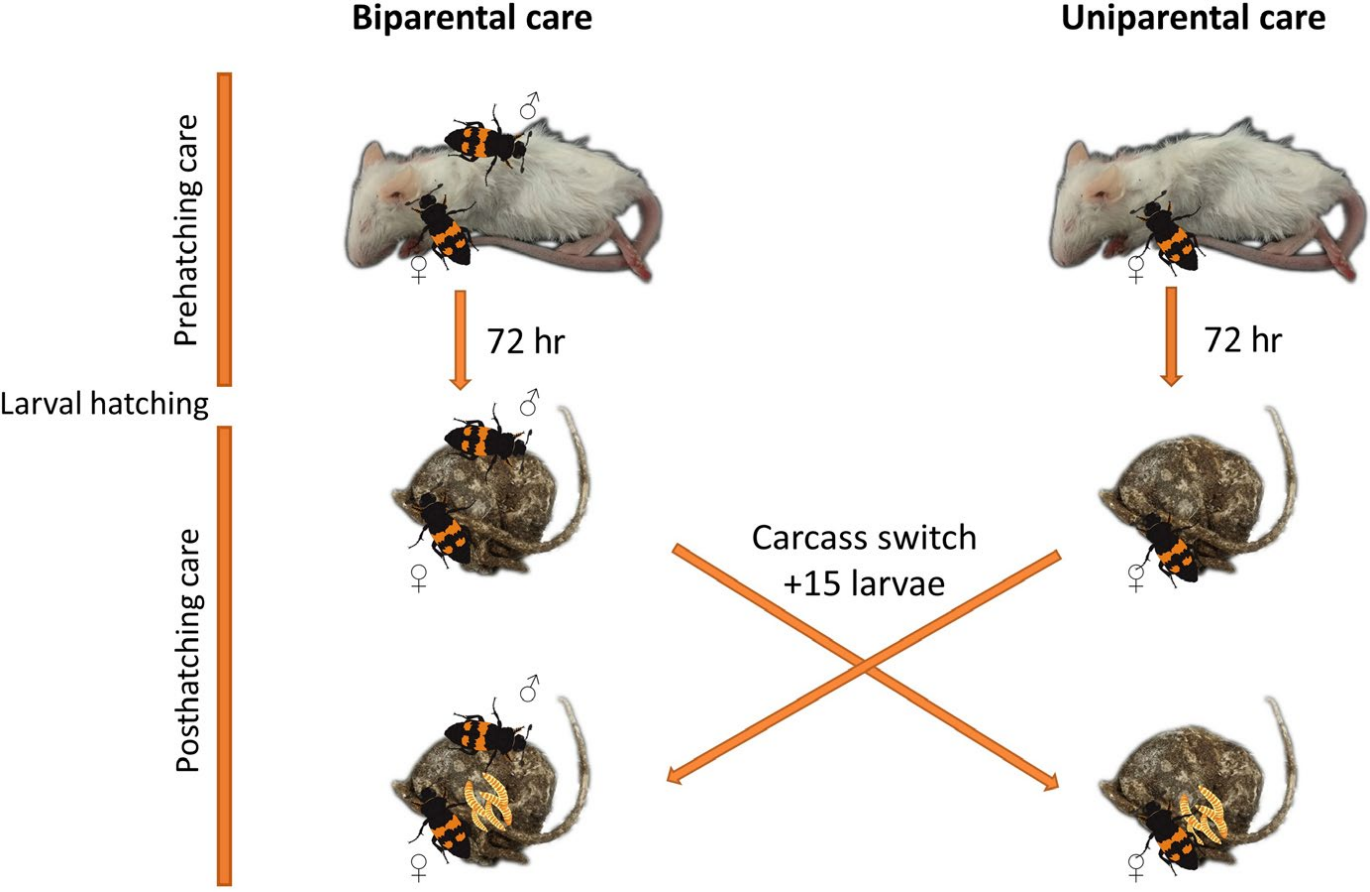
Illustration of the experimental design of the switch experiment. Pairs of beetles or females alone prepared an initially equally sized mouse carcass for roughly 72 hr. At the time of larval hatching, carcasses were switched between uni‐ and biparental parents and all broods received 15 larvae of mixed parentage

At 48 hr after the beetles had received the carcass, we started checking for eggs and transferred carcass and beetles into new containers if we spotted eggs at the bottom of the container. After 72 hr, shortly before the hatching of the larvae, we again transferred both carcass and beetles into a fresh container. However, at this point, we switched biparentally prepared carcasses with uniparental carcasses and vice versa (Figure 2). We chose this particular timepoint during the experiment because all of the concomitant differences in carcass size could only be caused by the different number of adults and not by the larvae. Of course, male parents continue eating even after larval hatching, but it is not possible to differentiate between larval and parental consumption from the carrion in the posthatching phase. We also started regularly checking for hatched larvae. We randomly provided each female or pair with 15 larvae of mixed parentage (see e.g., Capodeanu‐Nägler et al., 2018; Rauter & Moore, 2004) placed directly on the carcass. As parents reject larvae that arrive at the carcass too early, we started placing the larvae only after the parents' own offspring had started to hatch (Müller & Eggert, 1990). From that point on, we checked regularly for the desertion of parents and dispersal of larvae.

We excluded all broods that could not be provided with enough larvae, had lost a parent or failed to produce offspring. Our final sample size in the switch experiment consisted of *n* = 40 broods for 5 g carcasses (5 g carcass/biparental care *n* = 19; 5 g carcass/uniparental care *n* = 21) and *n* = 38 broods for 22 g carcasses (22 g carcass/biparental care *n* = 19; 22 g carcass/uniparental care *n* = 19).

#### 2.2.2 Control experiment: brood performance under uni‐ versus biparental care

To confirm the earlier studies (Bartlett, 1988; Müller et al., 1998; Smiseth et al., 2005) that females alone are equally successful in raising a brood compared with pairs of beetles providing that they are given the same amount of resources and the same number of larvae, we performed this additional experiment with a small carcass size that had not been tested for this assumption as yet. The experimental procedure and measurements were identical to the switch experiment described above, the only difference being the switching of carcasses within the same caring regime and not between caring regimes. Thus, pairs of beetles raised their brood on biparentally prepared carcasses and single females raised their brood on carcasses prepared by other single females. Carcass size in this experiment was 5.26 ± 0.19 g and did not differ between the uni‐ and biparental groups within this experiment (GLM: *F*_1,30_ = 0.47, *p* = .50) nor from the carcass size used in the 5g switch experiment (GLM: *F*_1,70_ = 0.27, *p* = .61). Sample size within this experiment consisted of *n* = 32 broods (*n* = 17 uniparental care; *n* = 15 biparental care).

#### 2.2.3 Mass change of beetles and carcasses and weight of larvae

In all experiments, we weighed the beetles before providing them with the carcasses, after 72 hr and at the time of desertion or, if a beetle did not desert the brood, at the time of larval dispersal. Carcasses were weighed at the beginning of the experiment and after 72 hr. Surviving larvae were counted and weighed at the time of dispersal. For all measurements, we used a precision scale (Kern ABJ 120‐4M, Kern und Sohn GmbH) and the values were measured to the nearest 0.0001 g (to 0.01 g for carcasses). We assessed the body size of beetles by measuring individual pronotum width with a digital calliper.

### 2.3 Statistical analyses

All data were analyzed by using R version 3.5.1 (R Core Team, 2016). Body size (pronotum width) of females did not differ between the uni‐ and biparental groups and was therefore excluded from further analysis (5 g switch: GLM: *F*_1,37_ = 0.006, *p* = .94; 22g switch: GLM: *F*_1,36_ = 1.69, *p* = .20; control experiment: GLM: *F*_1,30_ = 0.12, *p* = .73). To test for differences in the amount of carcass consumed during the first 72 hr, we conducted generalized linear models (GLM) with Gaussian error with the group (biparental or uniparental) as explanatory variables and the mass change of carcass as a response variable separately for the different experiments (5 g switch; 22 g switch; control experiment).

We also used GLMs to test for differences in brood performance, with total brood mass, average larval mass, and the number of surviving larvae as response variables and the group (bi‐ or uniparental) as explanatory variable. The number of surviving larvae was tested with a Poisson error structure and the other two variables with Gaussian error.

Additionally, we compared the total change in beetle body mass between the two sexes and the caring environments in the various experiments. We therefore created a parameter called “caring parent” containing sex and parental state, comprising the three possible forms “male—biparental,” “female—biparental,” and “female—uniparental.” Total change in beetle mass was obtained by subtracting pre‐reproductive mass from mass at desertion. We performed three GLMs with Gaussian error with the total weight change as the response variable and the caring parent as the explanatory variable, one for each experiment.

## 3 RESULTS

### 3.1 Mass change of uni‐ versus biparentally prepared carcasses

**Figure 3:**
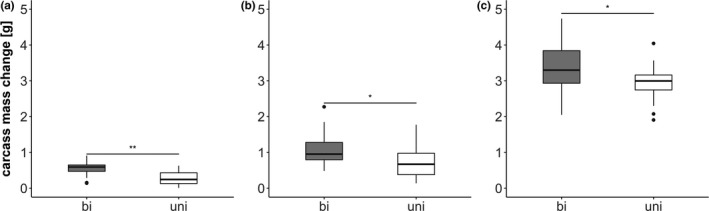
Amount of carcass mass reduced by the beetles in the first 72 hr. Gray bars represent biparental treatments, and white bars represent uniparental treatments. Boxplots show median, interquartile range, and minimum/maximum range. Points are values that fall outside the interquartile range (greater than 1.5 × interquartile range). a = 5 g control experiment; b = 5 g switch experiment; c = 22 g switch experiment. Asterisks indicate significant differences between uni‐ and biparental treatments (*p < .05, **p < .01)

In all three experiments, a pair of beetles reduced the weight of the carcass in the first 72 hr significantly more than a single female alone (control experiment: GLM: estimate: −0.26 ± 0.07, *t* = −3.45, *p* < .01, Figure 3a; switch 5 g: estimate: −0.33 ± 0.14, *t* = −2.04, *p* = .021 Figure 3b; switch 22 g: estimate: −0.47 ± 0.21, *t* = −2.27, *p* < .029, Figure 3c). Consequently, shortly before the larvae hatched, pairs had a slightly smaller carcass than single females in the control experiment and a slightly larger carcass than single females in the switch experiment.

### 3.2 Brood performance in the control and the switch experiment

**Figure 4:**
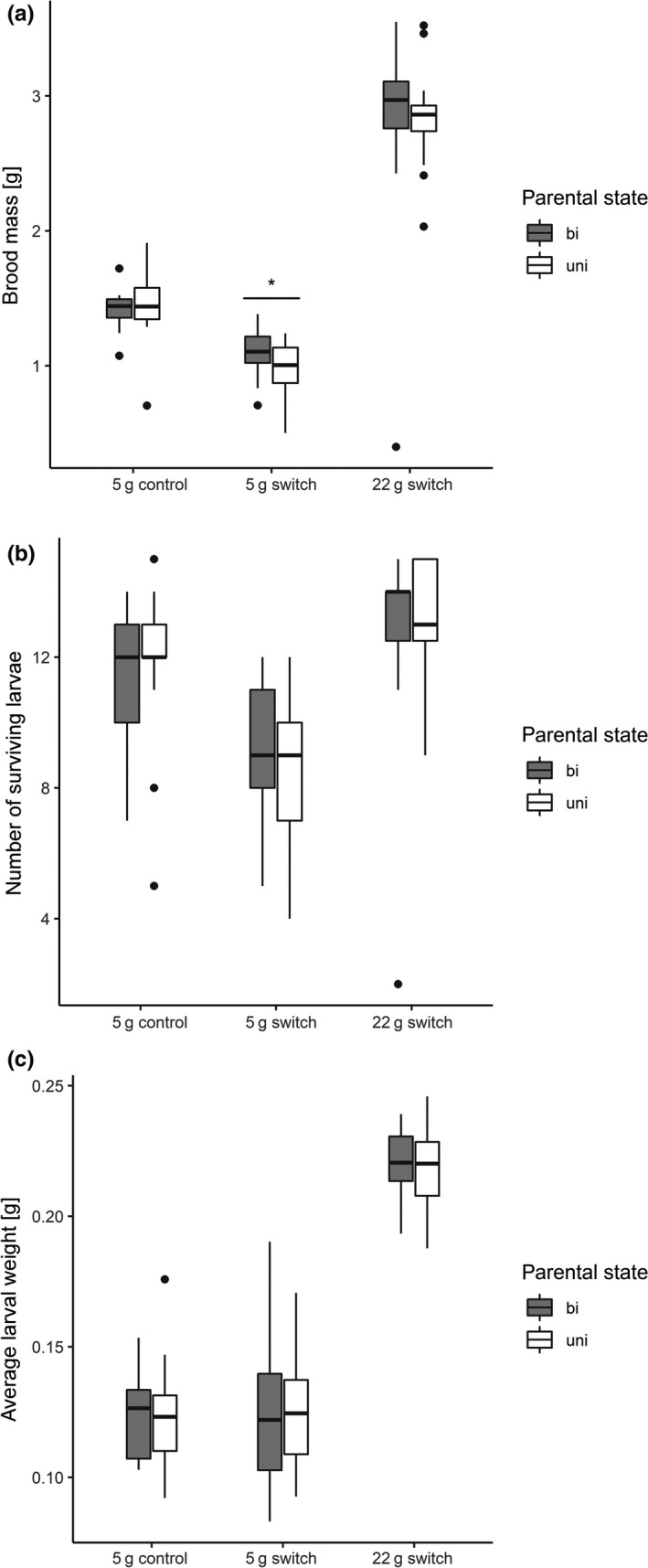
Results of offspring fitness measured at time of larval desertion (a: brood mass; b: number of surviving larvae; c: mean larval weight) in the various experiments (5 g control experiment, 5 g switch experiment, and 22 g switch experiment). Gray bars represent biparental treatments, and white bars represent uniparental treatments. Boxplots show median, interquartile range, and minimum/maximum range. Points are values that fall outside the interquartile range (greater than 1.5 × interquartile range). Asterisks indicate significant differences between uni‐ and biparental treatments (*p < .05)

As expected and similar to previous findings, no difference was found in brood performance between broods raised uniparentally on a uniparentally prepared carcass and broods raised with a male partner on a biparentally prepared carcass (control experiment). The total brood mass (estimate: 0.04 ± 0.08, *t* = 0.50, *p* = .62), number of surviving larvae (estimate: 0.02 ± 0.10, *z* = 0.225, *p* = .82), and the average larval weight (estimate: 0.0004 ± 0.006, *t* = 0.06, *p* = .95) showed no significant differences (Figure 4). Hence, even though the carrion resource lost more weight, if prepared under biparental condition (Figure 3; see result above), a female together with a male partner was able to raise an equal number and size of offspring to uniparental females.

However, if we switched the small 5 g carcasses after 72 hr between uni‐ and biparental treatments, biparental parents performed significantly better regarding total brood mass (estimate: −0.13 ± 0.06, *t* = −2.075, *p* = .045) but not with respect to the number of surviving larvae (estimate: −0.13 ± 0.11, *z* = −1.20, *p* = .23) or the average larval weight (estimate: −0.001 ± 0.008, *t* = −0.141, *p* = .88). Note that fewer larvae per brood survived in the 5 g switch experiment compared with the control experiment (Figure 4). Many factors are known to influence offspring fitness, from biotic ones like competition with mites (Gasperin, Duarte, & Kilner, 2015) or quantity and quality of the food (Hopwood, Moore, & Royle, 2014; Rebar, Leggett, Aspinall, Duarte, & Kilner, 2018; Rozen, Engelmoer, & Smiseth, 2008) or abiotic ones like temperature (Grew, Ratz, Richardson, & Smiseth, 2019). However, since we kept environmental factors, such as temperature, light condition, soil properties, and food resources constant among the treatments, we presently have no explanation for the discrepancy in larval fitness between the control and the 5 g switch experiment.

The switching of the larger 22 g carcasses between uni‐ and biparental treatments did not lead to a noticeable difference in brood performance between the uni‐ and biparental groups (brood mass: estimate: −0.001 ± 0.167, *t* = −0.006, *p* = .99; number of surviving larvae: estimate: 0.02 ± 0.09, *z* = 0.18, *p* = .86; average larval weight: estimate: −0.001 ± 0.005, *t* = −0.365, *p* = .72; Figure 4).

### 3.3 Weight change of beetles

Of a total of 163 beetles participating in all experiments, 156 beetles (males and females) gained weight in the prehatching phase of reproduction. The total change in body mass did not differ between the caring parents in the three experiments (control experiment: *F*_2,44_ = 0.46, *p* = .63; switch 5 g: *F*_2,56_ = 0.82, *p* = .44; switch 22 g: *F*_2,54_ = 0.02, *p* = .98).

## 4 DISCUSSION

The sharing of a resource between family members can lead to competition (Botterill‐James, Ford, While, & Smiseth, 2017; Kramer & Meunier, 2018), and it is not yet clear whether the benefits of a second caring male parent (Jenkins, Morris, & Blackman, 2000; Pilakouta et al., 2018) outweigh the drawbacks of a second eater at the limited food source (Keppner et al., 2018; Pilakouta et al., 2016).

Biparental care is thought to evolve when the net benefit for offspring fitness outweighs the costs of care for both parents (Gross, 2005; Trumbo, 2012). However, in some cases, caring biparentally is not the most favorable solution for all family members, as it might lead to competition. It is not entirely clear why male *Nicrophorus vespilloides* frequently participate in brood care. As pairs of beetles are more efficient in fighting off opponents (Eggert & Müller, 1997; Scott, 1990), a male staying with the female and the offspring to guard them might have been one of the first steps in the evolution of biparental care in *N. vespilloides*. While brood and mate guarding, the male also feeds from the carrion resource leaving the rest of the family with less food (Chemnitz et al., 2017; Keppner et al., 2018). Other behavioral traits such as offspring provisioning and carcass preparation by the male might have evolved as a mean to compensate for this loss of resource.

In our study, we have tried to shed light on whether assistance in brood care by male *Nicrophorus vespilloides* does have a positive effect on offspring fitness, but this effect is not easily demonstrable, as it is masked by the lack of food for the offspring because of the food consumption by the male from the shared resource. With our experiments, we first could confirm that the presence of a male had a negative effect on carcass mass, since the mass of the carrion resource was lower in pairs than in single females after the prehatching phase. Second, we found that despite of the lower amount of resources that was available for the larvae, pairs had an equal brood success than single females in our control experiment. However, switching the carcass between uniparental and biparental treatments at the end of the prehatching phase did lead to a difference in larval fitness. Two parents performed better and reared a heavier brood on a carcass prepared by a single female compared with a female beetle alone on a carcass prepared by two beetles. Hence, our results might provide evidence for compensation by the male parent, that is, they feed from the carrion resource, but this food loss is counterbalanced by their contribution to brood care. Below we discuss our results in more detail and consider their implications for the evolution of biparental care.

Burying beetles are known to gain body mass to a large extent during the first few days of reproduction (Keppner et al., 2018) and even food‐deprived beetles are able to replenish their lack of nutritional reserves within this short amount of time (Trumbo & Xhihani, 2015 studying *N. orbicollis*). Irrespective of parental mode and sex, 156 out of 163 beetles in our experiments gained weight during the first 72 hr after receiving a carcass. Consequently, almost all of the beetles must have consumed at least some food from the carcass while preparing it, as the carcass was the only food source available during this timeframe. We assume that the difference in weight reduction of a carcass prepared by either one or two beetles shows that they not only work to prepare the carcass for their soon‐to‐arrive offspring, but also feed on this valuable treat, with pairs of beetles consuming more than a single female alone.

Previous studies showed that uni‐ and biparental parents performed equally well when receiving an equal amount of carcass and caring for the same number of larvae (Bartlett, 1988; Müller et al., 1998; Smiseth et al., 2005), but not on the very small carcass size (5 g) that we have used. Indeed, in our control experiment we could find no differences in brood success and our results are therefore in line with the findings of the abovementioned studies. However, when considering our result that carcasses prepared by pairs lost more weight than carcasses of single mothers during the prehatching phase, it is rather surprising that single females were not able to raise a brood of larger mass than pairs. This result suggests that either the difference in carrion consumption is neglectable or that male care has somehow contributed to the growth of larvae, thereby outweighing the effect of carcass mass on brood mass. In fact, when we switched the differently prepared carcasses between the parental modes, pairs were able to produce a brood of larger mass than single females. Hence, these results indicate that the amount eaten is not trivial and has together with male care consequences for offspring growth. In another system, namely the European earwig *Forficula auricularia*, a caring mother that gained a considerable amount of weight had negative effects on the fitness of her clutch if food availability was restricted (Kramer et al., 2017). Studies on parent–offspring competition in the burying beetle *N. vespilloides* rendered variable results but this competition possibly occurs under biparental conditions (Gray, Richardson, Ratz, & Smiseth, 2018; Keppner et al., 2018; Pilakouta et al., 2016). As we stated in the introduction, we suggest that the results of this part of our study represent a link between the studies that found brood success to be equal between uni‐ and biparental conditions (Bartlett, 1988; Müller et al., 1998; Smiseth et al., 2005) and the recent work that has found two parents fare better than one (Pilakouta et al., 2018) in *N. vespilloides*. Our results hint that male burying beetles help with caring for offspring, leading to higher offspring fitness. However, this effect might be masked in studies comparing same carcass and clutch sizes, because males also consume the carcass, but becomes observable when the amounts of larvae and carcass are constant per parent or, as in our case, when carcasses are switched between pairs of beetles and single females after preparation. Certainly, the discrepancy in brood mass between our two treatments was very low. However, the reason for this small effect might be that males continue to feed from the carrion resource in the posthatching phase, that is, after the switch. A recent study has also shown that uniparental female care is more efficient under lower ambient temperatures compared with standard laboratory conditions (Grew et al., 2019). It might very well be possible that also the effects of a second caring parent might become more evident when environmental conditions are less favorable for the developing larvae. Additionally, we are not able to exclude the possibility that parents receiving a bigger carcass tend to increase the workload directed toward their offspring which might also explain an increase in brood mass.

Physiological costs of parental care, such as a loss in body weight, often arise from trade‐offs between caring for offspring and self‐investment (Alonso‐Alvarez & Velando, 2012). Contrary to our expectations, the mode of parental care did not influence the weight change of the beetles in any of the three experiments. This result does not support the findings of Pilakouta et al. (2018) who have reported that females gain more mass under biparental conditions. However, our uniparental treatment started at the time that the beetles received a carcass and not from the time of larval hatching as in the abovementioned study, a difference that might be the reason for the discrepancy in these results.

Interestingly, although much theoretical work has dealt with biparental care (see, for example, Gilbert & Manica, 2015; Houston, Székely, & McNamara, 2013; McNamara, 2003), little experimental evidence is available suggesting that two parents fare better at caring for offspring than a single parent (Pilakouta et al., 2018). However, biparental care has evolved as a seemingly stable strategy in a variety of taxa. Our results might explain male participation in brood care in the burying beetle *N. vespilloides,* but a positive effect of male care can only be seen when food is limited. When food is unlimited, males also show care behavior, such as feeding, but we have been unable to reveal any effect on brood performance. However, perhaps males cannot determine whether food is limited or plentiful and do not adjust the amount of work directed at their offspring based on this factor.

In summary, our results suggest a novel route for the evolution of biparental care behavior: male partners might have evolved to help in the care of offspring in order to compensate for their own food consumption. However, based on our current results, we cannot draw any conclusions about the sequence of trait evolution in *Nicrophorus* males. It might be possible that in a first step, males have evolved to stay with females to guard their brood and mates. Since a carcass is a nutrient rich resource, males benefitted from consuming parts, as this led to a higher sex pheromone production (Chemnitz et al., 2017). Subsequently, males might have evolved to participate actively in posthatching care as this compensated for their own food consumption leading to a higher brood mass in comparison with nonhelping males. However, this evolutionary scenario is only speculation at this point and future work (especially comparative studies) is needed. Furthermore, since the effect of our treatment was very small, it might be necessary to test our “compensation” hypothesis with different levels of competition between parents and offspring to be able to draw broader conclusions. For example, future studies could not only vary the size of the carcass but also the prebreeding nutritional condition of the males or the number of offspring the parents receive to raise.

## CONFLICT OF INTEREST

The authors declare that there is no conflict of interest regarding the publication of this article.

## AUTHOR CONTRIBUTIONS

E.M.K. and S.S. conceived the ideas and designed the experiments. E.M.K. performed the experiments, analyzed the data, and wrote the manuscript with contributions from S.S. All authors read and approved the final manuscript.

## Data Availability

Data associated with this work are available in the Dryad Digital Repository: https://doi.org/10.5061/dryad.j6q573n8z.
